# Factors Affecting Patients with Concurrent Deep Neck Infection and Cervical Necrotizing Fasciitis

**DOI:** 10.3390/diagnostics12020443

**Published:** 2022-02-09

**Authors:** Shih-Lung Chen, Shy-Chyi Chin, Yu-Chien Wang, Chia-Ying Ho

**Affiliations:** 1 Department of Otorhinolaryngology & Head and Neck Surgery, Chang Gung Memorial Hospital, Linkou 333, Taiwan; m7054@cgmh.org.tw; 2 School of Medicine, Chang Gung University, Taoyuan 333, Taiwan; b25chin@gmail.com (S.-C.C.); chiayingho23@gmail.com (C.-Y.H.); 3 Department of Medical Imaging and Intervention, Chang Gung Memorial Hospital, Linkou 333, Taiwan; 4 Department of Otorhinolaryngology & Head and Neck Surgery, New Taipei Municipal Tucheng Hospital, New Taipei City 236, Taiwan; 5 Division of Chinese Internal Medicine, Center for Traditional Chinese Medicine, Chang Gung Memorial Hospital, Taoyuan 333, Taiwan

**Keywords:** blood sugar, concurrent, cervical necrotizing fasciitis, C-reactive protein, deep neck infection, diabetes mellitus

## Abstract

Deep neck infection (DNI) is a severe disease of the deep neck spaces, which has the potential for airway obstruction. Cervical necrotizing fasciitis (CNF) is a fatal infection of the diffuse soft tissues and fascia with a high mortality rate. This study investigated risk factors in patients with concurrent DNI and CNF. A total of 556 patients with DNI were included in this study between August 2016 and December 2021. Among these patients, 31 had concurrent DNI and CNF. The relevant clinical variables were assessed. In univariate analysis, age (> 60 years, odds ratio (OR) = 2.491, *p* = 0.014), C-reactive protein (CRP, OR = 1.007, *p* < 0.001), blood sugar (OR = 1.007, *p* < 0.001), and diabetes mellitus (DM, OR = 4.017, *p* < 0.001) were significant risk factors for concurrent DNI and CNF. In multivariate analysis, CRP (OR = 1.006, *p* < 0.001) and blood sugar (OR = 1.006, *p* = 0.002) were independent risk factors in patients with concurrent DNI and CNF. There were significant differences in the length of hospital stay and therapeutic management (intubation, tracheostomy, incision and drainage) between DNI patients with and without CNF (all *p* < 0.05). While there were no differences in pathogens between the DNI alone and concurrent DNI and CNF groups (all *p* > 0.05), the rate of specific pathogen non-growth from blood cultures was 16.95% (89/525) in the DNI alone group, in contrast to 0% (0/31) in the concurrent DNI and CNF group (*p* = 0.008). Higher CRP and blood sugar levels were independent risk factors for the concurrence of DNI and CNF. With regard to prognosis, there were significant differences in the length of hospital stay and therapeutic management between the groups with and without CNF. While there were no significant differences in pathogens (all *p* > 0.05), no cases in the concurrent DNI and CNF group showed specific pathogen non-growth, in contrast to 89/525 patients in the group with DNI alone.

## 1. Introduction

Deep neck infection (DNI) is a life-threatening bacterial infection within the potential spaces of the deep cervical fascia [1,2]. It can lead to airway obstruction and cause severe morbidity, including severe cervical necrotizing fasciitis (CNF), severe sepsis, esophageal perforation, and descending necrotizing mediastinitis, and mortality [3,4,5,6,7,8]. CNF is a fulminant infection of the soft and connective tissues that spreads along the neck fascial planes and results in posterior venous and arterial thrombosis, followed by necrosis of the skin and other adjacent tissues [9,10].

Clinically, DNI is first suspected in patients with shortness of breath, localized heat, redness, and swelling in the neck. CNF presents as a rapid extensive infection with cervical erythema, tenderness, crepitus, respiratory distress, and sepsis [11]. Previous studies have shown that some patients may present with concurrent DNI and CNF [12,13]. Therapeutic management in patients with the coexistence of these two lethal diseases is complicated. This study was performed to investigate risk factors and prognostic variables for concurrent DNI and CNF.

## 2. Materials and Methods

**Figure 1:**
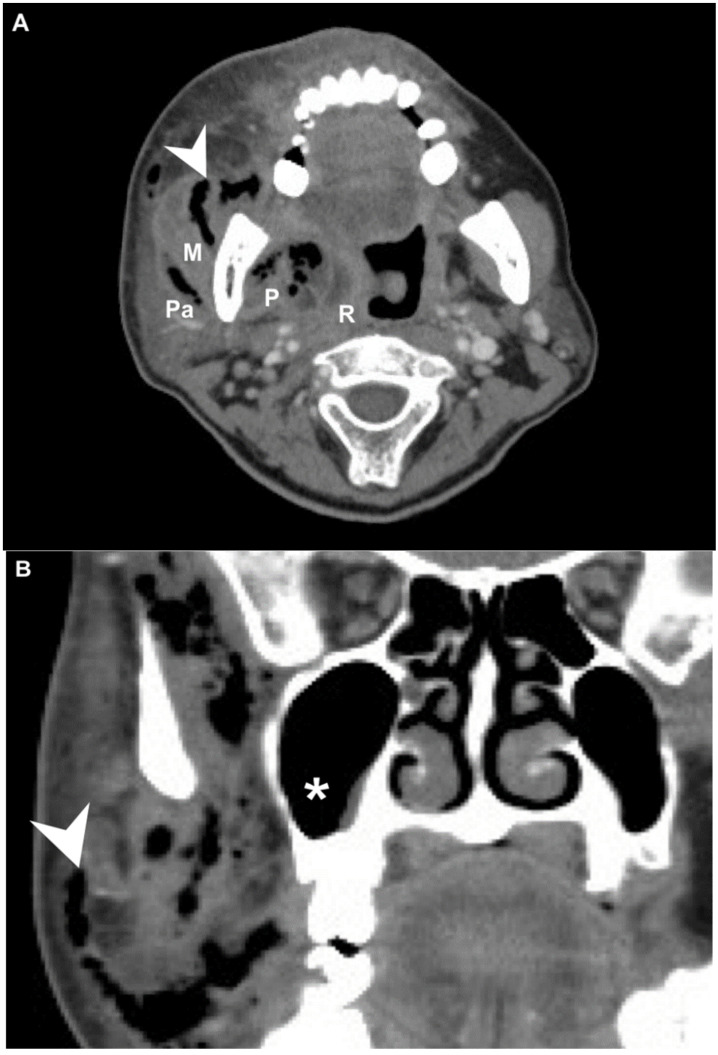
(**A**) Axial and (**B**) coronal view of a patient with concurrent deep neck infection and cervical necrotizing fasciitis on CT. Arrowhead: cervical necrotizing fasciitis; asterisk: maxillary sinus; P: parapharyngeal space; R: retropharyngeal space; M: masticator space; S: submandibular space.

We retrospectively reviewed the medical records of 556 patients diagnosed with DNI who were admitted to the Chang Gung Memorial Hospital in Linkou, a tertiary medical center in Taiwan, between August 2016 and December 2021. The diagnoses were performed by clinical presentation, ultrasonography (US) [12], and computed tomography (CT) (Figure 1A,B) [14]. Treatment included antibiotics, US-guided needle drainage, and open surgical incision and drainage. The empirical antibiotics used were ceftriaxone 1 gm Q12h and metronidazole 500 mg Q8h based on previous reports, in order to cover aerobic and anaerobic bacteria before the culture results were available [15,16,17].

### 2.1. Exclusion Criteria

Patients with severe cardiopulmonary diseases, previous head and neck tumor surgery, previous radiotherapy over the head and neck region, and mis-swallowing of a foreign body were excluded. A total of 556 patients with DNI were included in the study, among whom 31 had concurrent CNF.

### 2.2. Data Collection

To investigate the risk factors associated with concurrent DNI and CNF, we collected data on the patients’ sex, age, C-reactive protein (CRP) level, blood sugar level, and diabetes mellitus (DM) status, number of spaces involved in DNI, level of deep neck space involvement, presence of mediastinitis, length of hospital stay, intubation, tracheostomy, performance of incision and drainage (I&D) open surgery, and species of pathogens involved.

### 2.3. Ethics Statement

This study was approved on 10 January 2022 by the Institutional Review Board (IRB) of the Chang Gung Medical Foundation (IRB No.202200003B0). The data were collected retrospectively, and the patients were anonymized before data analysis. The IRB waived the need for informed consent.

### 2.4. Statistical Analysis

All data were analyzed using MedCalc software (ver. 18.6; MedCalc, Ostend, Belgium). As the Kolmogorov–Smirnov test showed that the data were not normally distributed, the chi-square test was used for categorical variables, the Mann–Whitney *U* test was used for comparison of continuous variables, and logistic regression analysis was used for the univariate and multivariate analyses. A multivariate forward stepwise selection procedure was implemented, and all of the variables included in the univariate analysis were entered into the final multivariate model. In all analyses, *p* < 0.05 was taken to indicate statistical significance.

## 3. Results

Demographic and clinical data are shown in Table 1. A total of 556 patients with DNI, consisting of 352 men (63.31%) and 204 women (36.69%), with a mean age of 52.66 ± 19.05 years, were included in the study. With regard to laboratory data, the mean CRP level was 155.76 ± 109.49 mg/L, and the mean blood sugar level was 149.50 ± 75.96 mg/dL. A total of 242 (43.52%) patients had DM.

A total of 195 (35.07%) patients exhibited the involvement of a single deep neck space, 169 (30.40%) exhibited the involvement of two spaces, and 192 (34.53%) exhibited the involvement of more than three spaces. Of those with deep neck space involvement, it involved the parapharyngeal space in 332 (59.71%) patients, the submandibular space in 251 (45.14%), the retropharyngeal space in 204 (36.69%), the masticator space in 115 (20.68%), the parotid space in 87 (15.64%), the anterior cervical space in 52 (9.35%), the visceral space in 47 (8.45%), the carotid space in 38 (6.83%), the perivertebral space in 25 (4.49%), and the posterior cervical space in 13 (2.33%). Mediastinitis was found in 77 (13.84%) patients. The mean length of hospital stay was 10.35 ± 8.43 days. Intubation was performed on 278 (50.00%) patients, and tracheostomy was performed on 102 (18.34%) patients. A total of 270 (48.56%) patients underwent I&D open surgery for DNI.

Table 1 lists the pathogens cultured from these patients. The overall rate of specific pathogen non-growth was 16.00% (89/556). Concurrent CNF was found in 31 (5.57%) patients.

Table 2 shows the results of the univariate analysis of variables for the 556 patients with DNI. The results show that age (>60 years, OR = 2.491, 95% confidence interval (CI): 1.184–5.241, *p* = 0.014), CRP (OR = 1.007, 95% CI: 1.004–1.010, *p* < 0.001), blood sugar (OR = 1.007, 95% CI: 1.004–1.011, *p* < 0.001), and DM (OR = 4.017, 95% CI: 1.764–9.148, *p* < 0.001) were significant risk factors for CNF. In Table 2, all factors were entered into a forward stepwise multivariate logistic regression model. CRP (OR = 1.006, 95% CI: 1.003–1.010, *p* < 0.001) and blood sugar (OR = 1.006, 95% CI: 1.002–1.010, *p* = 0.002) were significant independent risk factors for concurrent CNF in patients with DNI.

Table 3 shows a comparison of the management and length of hospital stay between the 31 patients with concurrent CNF and DNI and 525 patients with DNI alone. Significant differences were detected between the two groups in length of hospital stay (*p* = 0.001), intubation (*p* < 0.001), tracheostomy (*p* < 0.001), and I&D open surgery (*p* < 0.001).

As shown in Table 4, there were no significant differences in pathogens between the groups with and without CNF in DNI (all *p* > 0.05). However, there was no growth of specific pathogens from blood cultures in 16.95% (89/525) of patients with DNI alone, in contrast to none of the patients with concurrent DNI and CNF (*p* = 0.008).

## 4. Discussion

DNI can lead to severe life-threatening complications, while CNF is a potentially fatal emergent infection of the soft and connective tissues that may involve the rapid progression of edema, erythema, crepitus, tachycardia, hypotension, sepsis, and generalized necrosis of the superficial fascial layer and involved cutaneous tissue [10]. Management of concurrent DNI and CNF requires airway protection, including intubation and tracheostomy [11]. Further, broad-spectrum intravenous antibiotics and repeated aggressive debridement with removal of all necrotic tissue are necessary. In the present study, elevated CRP and blood sugar levels were independent risk factors associated with concurrent DNI and CNF. In addition, there were significant differences in the length of hospital stay and therapeutic management between the groups with and without CNF. While there were no differences in pathogens between the DNI alone and concurrent DNI and CNF groups, the rate of specific pathogen non-growth from blood cultures was 16.95% (89/525) in the DNI alone group in contrast to 0% (0/31) in the concurrent DNI and CNF group, and this difference was significant (*p* = 0.008). As both DNI and CNF can lead to life-threatening emergencies with potential airway compromise, early diagnosis is essential. DNI can be diagnosed by flexible fiberoscopy and CT, while there is no single gold standard for diagnosis of CNF [18]. Some groups consider CNF a surgical diagnosis confirmed by findings such as the presence of grayish necrotic fascia and the presence of foul-smelling pus [19]. However, other groups emphasize the importance of the patient’s clinical presentation as well as imaging findings [20,21]. In fact, the comprehensive diagnosis of concurrent DNI and CNF should be based not only on medical history and clinical symptoms but also on imaging examinations [22,23,24,25,26]. As shown in Table 2, age (>60 years), a higher CPR level, a higher blood sugar level, and DM were risk factors for concurrent DNI and CNF in the univariate analysis. Previous studies have reported that advanced age with CNF and DNI was associated with an increased risk of morbidity [27,28]. Our findings are consistent with those of Khamnuan et al., who reported that an age of >60 years significantly increased the mortality rate [29]. DM was another risk factor in the univariate analysis, while blood sugar was an independent risk factor in both the univariate and multivariate analyses for the concurrence of DNI and CNF. Cheng et al. [30] considered DM patients to have an increased susceptibility to necrotizing fasciitis, and DM has been reported to be a common underlying disease in these patients, present in >40% of cases. They reported that DM patients were significantly older than non-DM patients at the onset of necrotizing fasciitis. The presence of DM significantly affects clinical outcomes and is associated with longer hospital stays and a higher incidence of complications [31]. This is because DM leads to impaired cutaneous wound healing and an increased susceptibility to infection, which may affect the course of soft tissue infections [32]. A high blood sugar level is associated with complications from multiple spaces of DNI, and Zheng et al. [33] reported that >50% of DM patients in their study population had complications, which was significantly higher than the rate for the non-DM group. Sideris et al. reported that CRP and blood sugar levels are highly correlated, and they considered the blood sugar level to be one of the most important clinical variables [18]. Due to the increased incidence of high blood sugar observed in patients with concurrent CNF and DNI, we suggest that the blood sugar level should be monitored initially and controlled after admission. Repeated surgical debridement and drainage are essential in the management of concurrent DNI and CNF. In our cohort, the length of hospital stay and rates of intubation, tracheostomy, and I&D open surgery were higher in the group with concurrent DNI and CNF than the DNI alone group. Tracheostomy was performed in 17 (54.83%) patients with concurrent CNF and DNI. The involvement of multiple deep neck spaces was previously reported to be a risk factor for requiring tracheostomy [27]. For surgery, complete debridement, such as facial-cervical fasciotomy, should be performed with removal of all involved tissue, including the pharyngeal wall, larynx, cranial nerves, and bone. Serial wound debridement is suggested to avoid the development of severe sepsis [34]. However, repeated surgery results in major soft tissue defects. Intensive debridement wound care with wet dressing changes at least 4–6 times a day is recommended. A strategy of watchful waiting would prolong the time to elimination of the disease, which would lead to increased morbidity and a reduced survival rate. Reconstruction should be performed after removal of necrotizing tissue, where healthy granulating tissue is present in the wound bed with complete infection control [11]. Both CNF and DNI are usually polymicrobial in origin and include an admixture of aerobic and anaerobic species [35]. Patients were initially treated with empirical, broad-spectrum, intravenous antibiotics, which were then narrowed according to the results of wound culture. The pathogenesis of CNF begins with the entry of organisms or spores into the soft tissues, and then deeper tissues become infected, larger venules and arterioles are occluded, and necrosis affects all tissue layers [18]. Infections of the second and third molars of the mandible are the most frequent etiologies of odontogenic CNF [36,37]. Similarly, most cases of DNI are of odontogenic origin, and less common causes include peritonsillar abscesses, epiglottitis, and penetrating injuries of the mouth [38]. Odontogenic infections also contribute by indirect spread to adjacent tissue or deep neck spaces [39,40]. Hence, when odontogenic DNI penetrates into the deep neck spaces and spreads toward the skin, these deeper subcutaneous connective tissues are infected, followed by facial plane infection, causing concurrent DNI and CNF. As shown in Table 4, there were no significant differences in pathogens between the patients with concurrent DNI and CNF and the patients with DNI alone. Ferzli et al. reported that Group A *Streptococcus* was present in the majority of their patients [11]. Some studies have reported that CNF usually involves mixed flora, such as *Prevotella* and aerobes, mostly *Streptococcus* and *Staphylococcus aureus* [35,41]. For DNI, aerobic and anaerobic organisms reflect the oropharyngeal microbial community [42]. In our cohort, *Streptococcus constellatus* was the most commonly cultivated pathogen in the DNI patients regardless of whether they had concurrent CNF (19.35%) or DNI alone (19.42%). This microorganism is a small catalase-negative coccus belonging to the Anginosus group, along with *Streptococcus anginosus* and *Streptococcus intermedius* [43]. In this study, the overall rate of specific pathogen non-growth was 16.00% (89/556). Blood culture is not a highly sensitive method for identifying pathogens, especially when antibiotics have already been administered [44,45]. However, while there were no differences in pathogens between the DNI alone and concurrent DNI and CNF groups, the rate of specific pathogen non-growth from blood cultures was 16.95% (89/525) in the DNI alone group, in contrast to 0% (0/31) in the concurrent DNI and CNF group, suggesting that concurrent DNI and CNF is more serious and more difficult to manage than DNI alone.

### Limitations of the Study

This study had some limitations. First, the retrospective nature of the study resulted in a certain attrition rate. In addition, most patients were male, which could have been due to selection bias.

## 5. Conclusions

Higher CRP and blood sugar levels were independent risk factors associated with concurrent DNI and CNF. We suggest that the blood sugar level should be monitored initially and controlled after admission. For patients >60 years old with concurrent DNI and CNF, physicians should pay more attention to care because both diseases are fatal illnesses. With regard to prognosis, because concurrent DNI and CNF is more serious and more difficult to manage than DNI alone, there were significant differences in the length of hospital stay and therapeutic management between the DNI groups with and without concurrent CNF. While there were no differences in pathogens between the two groups, there were no cases in the concurrent DNI and CNF group with specific pathogen non-growth from blood cultures, in contrast to a rate of 16.95% in the DNI alone group.

## Figures and Tables

**Table 1:** Clinical characteristics of the 556 patients with DNI.

Characteristics	N (%)
Gender	556 (100.00)
Male	352 (63.31)
Female	204 (36.69)
Age, years (SD)	52.66 ± 19.05
CRP, mg/L (SD)	155.76 ± 109.49
Blood sugar, mg/dL (SD)	149.50 ± 75.96
Diabetes mellitus	242 (43.52)
Number of deep neck spaces involved	
Single space	195 (35.07)
Two spaces	169 (30.40)
Multiple spaces, ≥3	192 (34.53)
Deep neck space involvement	
Parapharyngeal space	332 (59.71)
Submandibular space	251 (45.14)
Retropharyngeal space	204 (36.69)
Masticator space	115 (20.68)
Parotid space	87 (15.64)
Anterior cervical space	52 (9.35)
Visceral space	47 (8.45)
Carotid space	38 (6.83)
Perivertebral space	25 (4.49)
Posterior cervical space	13 (2.33)
Mediastinitis	77 (13.84)
Length of hospital stay, days (SD)	10.35 ± 8.43
Intubation	278 (50.00)
Tracheostomy	102 (18.34)
Incision and drainage open surgery	270 (48.56)
Pathogens	
*Streptococcus constellatus*	108 (19.42)
*Parvimonas micra*	67 (12.05)
*Prevotella intermedia*	67 (12.05)
*Klebsiella pneumonia*	63 (11.33)
*Streptococcus anginosus*	61 (10.97)
*Prevotella buccae*	58 (10.43)
*Staphylococcus aureus*	34 (6.11)
*Streptococcus salivarius*	23 (4.13)
*Staphylococcus epidemidis*	21 (3.77)
*Streptococcus oralis*	18 (3.23)
*Gemella morbillorum*	17 (3.05)
*Eikenella corrodens*	16 (2.87)
*Salmonella enterica*	14 (2.51)
*Slackia exigua*	11 (1.97)
*Pseudomonas aeruginosa*	7 (1.25)
No growth	89 (16.00)
Cervical necrotizing fasciitis	31 (5.57)

DNI = deep neck infection; N = numbers; SD = standard deviation; CRP = C-reactive protein (normal range < 5 mg/L); blood sugar (normal range: 70–100 mg/dL).

**Table 2:** Univariate and multivariate analyses of 525 patients with DNI alone compared to 31 patients with concurrent DNI and CNF.

Variable	CNF		Univariate Analysis			Multivariate Analysis		
	Yes	No	OR	95% CI	*p* Value	OR	95% CI	*p* Value
Gender	31	525			0.533			
Male	18	334	0.791	0.479–2.085				
Female	13	191	1.000					
Age, years					**0.014** *	-	-	-
>60	19	204	2.491	1.184–5.241				
≤60	12	321	1.000					
CRP, mg/L (SD)	246.77 ± 67.62	150.38 ± 109.16	1.007	1.004–1.010	**<0.001** *	1.006	1.003–1.010	**<0.001** *
Blood sugar, mg/dL (SD)	216.41 ± 55.56	145.55 ± 75.19	1.007	1.004–1.011	**<0.001** *	1.006	1.002–1.010	**0.002** *
Diabetes mellitus					**<0.001** *	-	-	-
Yes	23	219	4.017	1.764–9.148				
No	8	306	1.000					
Multiple spaces, ≥3					0.136			
Yes	7	185	0.536	0.226–1.267				
No	24	340	1.000					
Parapharyngeal space					0.082			
Yes	23	309	2.009	0.439–2.277				
No	8	216	1.000					
Submandibular space					0.063			
Yes	19	232	1.999	0.951–4.203				
No	12	293	1.000					
Retropharyngeal space					0.184			
Yes	8	196	0.583	0.256–1.330				
No	23	329	1.000					
Masticator space					0.132			
Yes	3	112	0.395	0.117–1.323				
No	28	413	1.000					
Parotid space					0.164			
Yes	2	85	0.357	0.083–1.524				
No	29	440	1.000					
Anterior cervical space					0.253			
Yes	1	51	0.309	0.041–2.319				
No	30	474	1.000					
Visceral space					0.152			
Yes	5	42	2.211	0.807–6.058				
No	26	483	1.000					
Carotid space					0.930			
Yes	2	36	0.936	0.214–4.083				
No	29	489	1.000					
Perivertebral space					0.591			
Yes	2	23	1.505	0.338–6.696				
No	29	502	1.000					
Posterior cervical space					0.191			
Yes	2	11	3.222	0.682–15.21				
No	29	514	1.000					
Mediastinitis					0.382			
Yes	6	71	1.534	0.608–3.872				
No	25	454	1.000					

DNI = deep neck infection; CNF = cervical necrotizing fasciitis; SD = standard deviation; OR = odds ratio; CI = confidence intervals; CRP = C-reactive protein; *, *p* < 0.05. Significant differences are shown in bold.

**Table 3:** Comparison of management and length of hospital stay between 525 patients with DNI alone compared to 31 patients with concurrent DNI and CNF.

Characteristics	CNF, N = 31 (%)	Non-CNF, N = 525 (%)	*p* Value
Length of hospital stay, days (SD)	12.61 ± 5.79	10.21 ± 8.55	**0.001** *
Intubation			**<0.001** *
Yes	30 (96.77)	248 (47.23)	
No	1 (3.23)	277 (52.77)	
Tracheostomy			**<0.001** *
Yes	17 (54.83)	85 (16.19)	
No	14 (45.17)	440 (83.81)	
I&D open surgery			**<0.001** *
Yes	30 (96.77)	240 (45.71)	
No	1 (3.23)	285 (54.29)	

DNI = deep neck infection; CNF = cervical necrotizing fasciitis; N = number; I&D = incision and drainage; *, *p* < 0.05. Significant differences are shown in bold.

**Table 4:** Comparison of pathogens between 525 patients with DNI alone compared to 31 patients with concurrent DNI and CNF.

Pathogens	CNF, N = 31 (%)	Non-CNF, N = 525 (%)	*p* Value
*Streptococcus constellatus*	6 (19.35)	102 (19.42)	0.992
*Parvimonas micra*	3 (9.67)	64 (12.19)	1.000
*Prevotella intermedia*	4 (12.90)	63 (12.00)	0.780
*Klebsiella pneumonia*	5 (16.12)	58 (11.04)	0.391
*Streptococcus anginosus*	5 (16.12)	56 (10.66)	0.369
*Prevotella buccae*	3 (9.67)	55 (10.47)	1.000
*Staphylococcus aureus*	4 (12.90)	30 (5.71)	0.112
*Streptococcus salivarius*	3 (9.67)	20 (3.80)	0.130
*Staphylococcus epidemidis*	3 (9.67)	18 (3.42)	0.105
*Streptococcus oralis*	3 (9.67)	15 (2.85)	0.072
*Gemella morbillorum*	1 (3.22)	16 (3.04)	1.000
*Eikenella corrodens*	2 (6.45)	14 (2.66)	0.222
*Salmonella enterica*	2 (6.45)	12 (2.28)	0.180
*Slackia exigua*	2 (6.45)	9 (1.71)	0.120
*Pseudomonas aeruginosa*	1 (3.22)	6 (1.14)	0.332
No growth	0 (0.00)	89 (16.95)	**0.008** *

DNI = deep neck infection; N = number; CNF = cervical necrotizing fasciitis; *, *p* < 0.05. Significant differences are shown in bold.

## Data Availability

All data generated or analyzed during this study are included in this published article. The data are available on request.

## Abbreviations

CNF: cervical necrotizing fasciitis; CT: computed tomography; CRP: C-reactive protein; CI: confidence interval; DNI: deep neck infection; DM: diabetes mellitus; US: ultrasonography; OR: odds ratio.
